# Clinical practice and outcome of patients with elderly‐onset ulcerative colitis: Insights from a nationwide claims database study in Japan

**DOI:** 10.1002/jgh3.13103

**Published:** 2024-06-17

**Authors:** Rintaro Moroi, Yoichi Kakuta, Hiroshi Nagai, Yusuke Shimoyama, Takeo Naito, Hisashi Shiga, Yoshitaka Kinouchi, Atsushi Masamune

**Affiliations:** ^1^ Division of Gastroenterology Tohoku University Graduate School of Medicine Sendai Japan; ^2^ Student Healthcare Center, Institute for Excellence in Higher Education Tohoku University Sendai Japan

**Keywords:** claims database, molecular targeting drug, older onset, steroid, ulcerative colitis

## Abstract

**Background and Aim:**

The number of older patients with ulcerative colitis is increasing; however, limited data exist regarding the differences between elderly‐ and non‐elderly‐onset ulcerative colitis. We aimed to compare the clinical practice and course of elderly‐onset ulcerative colitis with those of non‐elderly‐onset ulcerative colitis.

**Methods:**

We selected older patients with ulcerative colitis and divided them into the elderly‐ and non‐elderly‐onset ulcerative colitis groups according to their age at onset. We compared the cumulative systemic steroid‐free, molecular targeting drug‐free, and surgery‐free rates between the two groups. We performed a multivariate analysis to identify the clinical factors related to systemic steroid administration, the use of molecular targeting drugs, surgery, and death.

**Results:**

We collected data of 2669 and 277 elderly and non‐elderly‐onset ulcerative colitis patients, respectively. The cumulative systemic steroid‐free rate of elderly‐onset ulcerative colitis was significantly lower than that of non‐elderly‐onset ulcerative colitis. However, no difference was observed in the cumulative molecular targeting drugs and surgery‐free rates between the two groups. Elderly‐onset ulcerative colitis significantly increased the risk of systemic steroid administration and death but not the use of molecular targeting drugs and surgery.

**Conclusion:**

The disease severity of ulcerative colitis and clinical practice may not differ between the elderly‐ and non‐elderly‐onset groups. However, elderly‐onset ulcerative colitis was associated with increased mortality risk. Thus, we need to pay attention to the patients' condition and appropriate timing of surgery for patients with elderly‐onset ulcerative colitis.

## Graphical abstract

The severity and clinical course between the elderly onset and non‐elderly onset Ulcerative colitis groups may not differ in the long term after onset of ulcerative colitis.

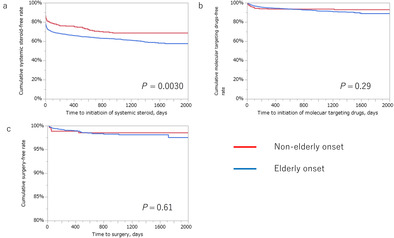

## Introduction

Ulcerative colitis (UC) is an inflammatory bowel disease characterized by chronic inflammation of the colon with repeated exacerbation and remission.^1^, ^2^, ^3^ Recently, the number of patients diagnosed with UC has been increasing.^4^ Japan is an aging society, with the population aged ≥65 years of 36 236 000 (29.0% of the total population).^5^ As an aging society progresses, the number of older patients with UC is expected to increase. The first peak of UC onset is at 20–30 years, and the second peak is observed in the older age.^6^, ^7^, ^8^ Consequently, the number of older patients with UC is increasing in various countries,^9^, ^10^ including Japan.^11^, ^12^

Older patients with UC are divided into two types based on their age at onset: (i) patients who develop UC in older ages (elderly‐onset UC [EOUC]) and (ii) those who develop UC at a younger age and become older (non‐elderly‐onset UC [NEOUC]). However, limited data exist regarding the differences between EOUC and NEOUC. Older patients with UC generally require careful treatments than younger patients because of frailty and high risk of complications. Therefore, clarifying the clinical practices for older patients and differences between EOUC and NEOUC is essential.

The number of older patients with UC in a single center is relatively small to analyze; thus, a single‐center study may be inadequate. On the contrary, a nationwide database, the so‐called big data, provides an adequate number of samples and enables the analysis of rare diseases and situations, such as those previously reported.^13^, ^14^, ^15^, ^16^, ^17^, ^18^, ^19^, ^20^ Therefore, this study aimed to clarify the clinical practice of EOUC and compare the differences in clinical course between EOUC and NEOUC using a nationwide claims database in Japan.

## Methods

### Data source

In this study, we contracted DeSC Healthcare, Inc. (https://desc-hc.co.jp/en), a joint company providing various healthcare services, and obtained permission to use their dataset comprising the prescription claim receipts of patients with UC. This dataset contains anonymized inpatient and outpatient prescription claims records, which include age, sex, medical treatment, surgery, and clinical diagnosis, based on the International Classification of Diseases 10th version (ICD‐10) from a health insurance association in Japan from April 2014 to February 2022.

### Extraction of eligible patients and data collection

**Figure 1:**
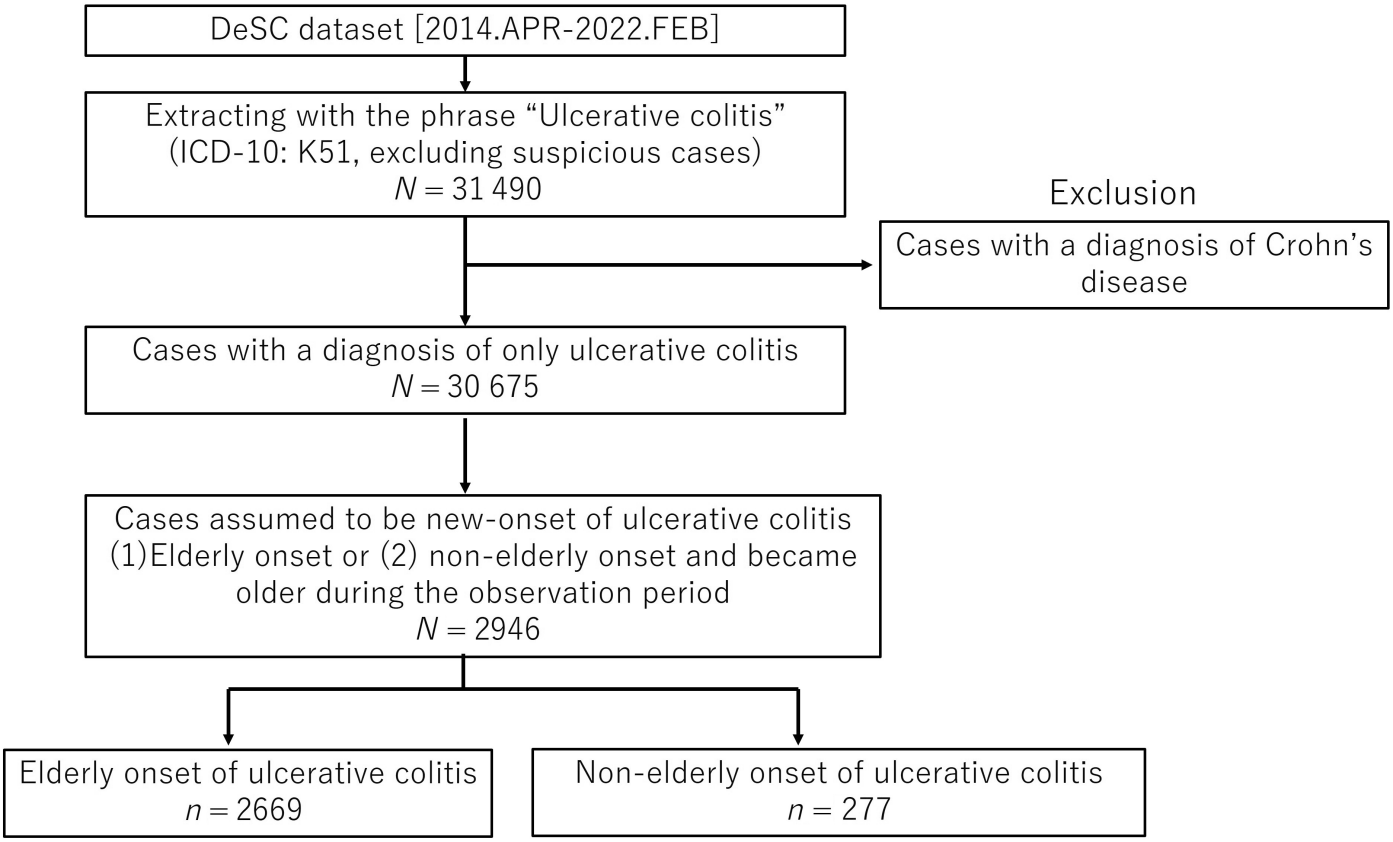
The flowchart for extraction of eligible patients from the patient claims database of DeSC Healthcare, Inc.

Figure 1 shows the selection flowchart of eligible patients from the DeSC Healthcare database. We identified UC cases using ICD‐10 code K51. We excluded the following cases: (i) suspicious cases containing the word “suspicious” and (ii) cases that had Crohn's disease in their diagnosis. Subsequently, we extracted cases that were assumed to have newly developed UC based on the definitions described below. The DeSC dataset provided the data on the patients' birth year and month, which enabled the estimation of patients' age at UC onset. We eventually selected patients aged ≥65 years at the end of the observation period and divided the eligible patients into two groups as follows: (i) patients who developed UC at age ≥65 years (elderly‐onset [EO] group) and (ii) those who developed UC at age <65 years and became aged ≥65 years during the observation period (non‐elderly‐onset [NEO] group). The definition of elderly is ≥65 years based on the World Health Organization classification.^21^

We collected the following patient data from the DeSC dataset: sex, start and end dates of observation, and surgery. Information regarding prescribed medications including 5‐aminosalicylic acid (5‐ASA), systemic steroid administration (prednisolone), immunomodulators (azathioprine and 6‐mercaptopurine), molecular targeting drugs (MTDs) including biologics (infliximab, adalimumab, golimumab, ustekinumab, and vedolizumab) and small‐molecule agents (tofacitinib, tacrolimus, and cyclosporine) were also collected.

### Definitions of new prescription and new development of UC

The definitions described below are similar with those of our previous study.^22^ We defined a “new” prescription for each drug as no prescription within 26 weeks before the first prescription date of the medicine during the observation period. Other types of prescriptions were defined as “old” prescriptions.

Prescription was defined as discontinuation in cases of no prescription for >13 weeks from the next scheduled prescription date. The next scheduled prescription date differed between drugs. For instance, the next scheduled prescription date for ustekinumab was 12 weeks after the last prescription date (infliximab and vedolizumab were 8 weeks, adalimumab and golimumab were number of prescriptions × 14 days, and other drugs were prescription days).

We also assumed cases in our study that had both (i) new prescription of either 5‐ASA, systemic steroid, or topical steroid drugs and (ii) no old prescription of such drugs during the observation period as new‐onset UC. Based on this assumption, we estimated the age at UC onset.

### Data and statistical analyses

Geographical data, medications including the number of MTD use and systemic steroid prescription days, and clinical event (surgery and death) rates were compared between the EO and NEO groups using the chi‐square test. We compared the cumulative surgery‐free, systemic steroid‐free, MTD‐free, and cumulative treatment persistence rates of the first MTD between the EO and NEO groups using the Kaplan–Meier method. We also conducted a comparative analysis of the efficacy of combination therapy involving infliximab and azathioprine, focusing on the treatment persistence rate as assessed through the Kaplan–Meier method in both EO and NEO groups. Combination therapy was defined as the confirmation of azathioprine prescription concurrent at the initiation of infliximab.

Multivariate logistic regression analysis was performed to identify the clinical factors related to systemic steroid administration, use of MTDs, surgery, and death. Statistical significance was set at *P* < 0.05. All analyses were performed using the JMP Pro17 software (SAS Institute, Tokyo, Japan).

### Ethics approval and patient consent statement

The study protocol was reviewed and approved by the Ethics Committee of the Tohoku University Graduate School of Medicine (2022‐1‐412). The requirement for informed consent was waived because of the anonymity of patient data.

## Results

### Background of the study population

We extracted the data of 2946 older patients with UC, of whom 2669 were assigned to the EO group and 277 to the NEO group. Patient characteristics are summarized in Table 1. The mean observation period was 1629.6 days. The administration rates of systemic steroids and MTD were 34.2% (1008 of 3946) and 7.0% (205 of 2669), respectively. The surgery rate was 2.2% (65 of 2946).

**Table 1:** Background of older patients with UC

	Older patients with UC *n* = 2946
Sex (male/female)	1664/1282
Age categories of UC onset	
Elderly onset	2.669
Non‐elderly onset	277
Average observation period (SD)	1629.6 (633.7) days
Academic hospital	16 (0.54%)
Use of systemic steroid	1008 (34.2%)
Use of molecular targeting drug	205 (7.0%)
Breakdown of the first molecular targeting drug	
Infliximab	68
Adalimumab	17
Golimumab	12
Ustekunumab	13
Vedolizumab	37
Tofacitinib	4
Tacrolimus	40
Cyclosporine po	13
Cyclosporine iv	1
Surgery	65 (2.2%)
Death	121 (4.1%)

iv, intravenous injection; po, per os; UC, ulcerative colitis.

### Comparison of geographical data, medications, and clinical event rates between the elderly‐ and non‐elderly‐onset groups

Table 2 shows the comparison between the EO and NEO groups. No significant differences were observed in the male/female ratio, academic hospital rate, administration rates of systemic steroids and MTDs, the number of MTD use, prescription days of systemic steroid, or surgery rate between the two groups. Furthermore, no difference was observed in the number of MTDs between the EO and NEO groups. The rate of infliximab as the first MTD in the EO group was lower than that in the NEO group (31.4% *vs* 50.0%), whereas vedolizumab was more frequently selected in the EO group compared with the NEO group (19.5% *vs* 5.0%). The mortality rate in the EO group was higher than that in the NEO group (4.5% *vs* 0.72%, *P* = 0.0011).

**Table 2:** Comparisons of backgrounds, treatment, and outcomes between elderly‐ and non‐elderly‐onset UC

	Non‐elderly onset *n* = 277	Elderly onset *n* = 2669	*P*‐value
Sex (male/female)	145/132	1519/1150	0.16
Average age at the end of observation (SD)	67.0 (1.7) years	77.4 (7.3) years	**<0.0001**
Academic hospital	2 (0.72%)	14 (0.52%)	0.66
Use of systemic steroid	84 (30.3%)	924 (34.6%)	0.16
Median prescription days of systemic steroid through po (inter‐quartile range)	70 days (11–245)	61 days (9–213)	0.72
Median prescription days of systemic steroid through iv (inter‐quartile range)	14 days (3–27)	10 days (4–22)	0.53
Use of molecular targeting drug	20 (7.2%)	185 (6.9%)	0.80
Breakdown of the first molecular targeting drug			
Infliximab	10 (50.0%)	58 (31.4%)	
Adalimumab	3 (15.0%)	14 (7.6%)	
Golimumab	1 (5.0%)	11 (6.0%)	
Ustekunumab	0 (0%)	13 (7.0%)	
Vedolizumab	1 (5.0%)	36 (19.5%)	
Tofacitinib	1 (5.0%)	3 (1.6%)	
Tacrolimus	4 (20.0%)	36 (19.5%)	
Cyclosporine po	0 (0%)	13 (7.0%)	
Cyclosporine iv	0 (0%)	1 (0.5%)	
The number of molecular targeting drug use			0.56
0	257 (92.8%)	2484 (93.1%)	
1	13 (4.7%)	140 (5.3%)	
≥2	7 (2.5%)	45 (1.7%)	
Surgery	5 (1.8%)	60 (2.3%)	0.83
Death	2 (0.72%)	119 (4.5%)	**0.0011**

The bold value means statistical significance.

iv, intravenous injection; po, per os; UC, ulcerative colitis.

### Differences in long‐term prognosis after onset between the elderly‐ and non‐elderly‐onset groups

**Figure 2:**
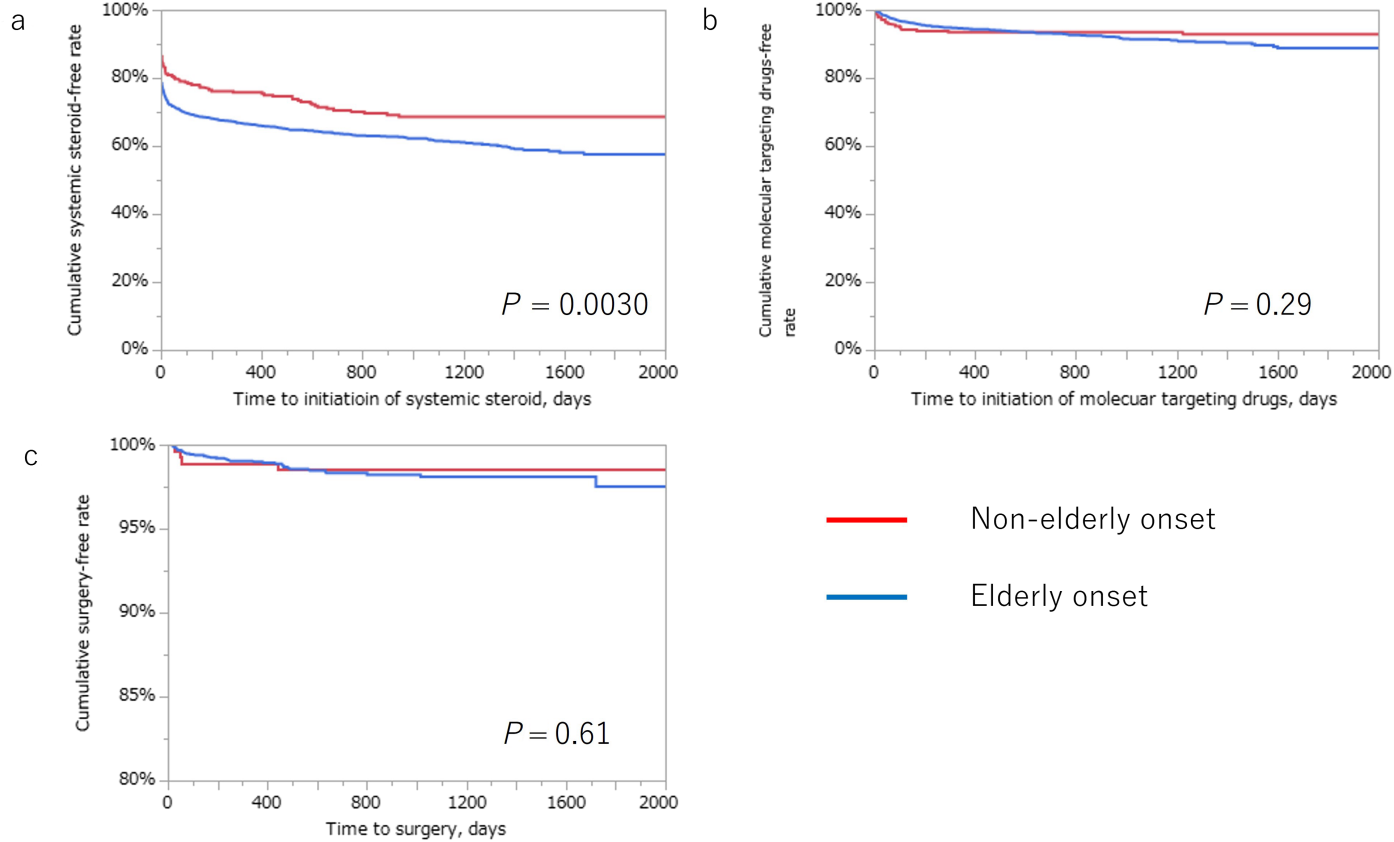
The Kaplan–Meier curve describing the differences in long‐term prognosis between onset age categories. (a) The cumulative systemic steroid‐free rates of elderly‐ and non‐elderly‐onset ulcerative colitis at 5 years are 57.6% and 68.6%, respectively (*P* = 0.0030). (b) The cumulative molecular targeting drug‐free rates of elderly‐ and non‐elderly‐onset ulcerative colitis at 5 years are 88.8% and 92.9%, respectively (*P* = 0.29). (c) The cumulative surgery‐free rates of elderly‐ and non‐elderly‐onset ulcerative colitis at 5 years are 97.5% and 98.5%, respectively (*P* = 0.29).

The cumulative systemic steroid‐free, MTD‐free, and surgery‐free rates in the EO and NEO groups are shown in Figure 2. The cumulative systemic steroid‐free rate at 5 years after onset in the EO group was significantly lower than that in the NEO group (57.6% *vs* 68.6%, *P* = 0.0030), whereas no significant difference was observed in the cumulative MTDs or surgery‐free rates between the two groups (*P* = 0.29 and *P* = 0.61, respectively).

**Figure 3:**
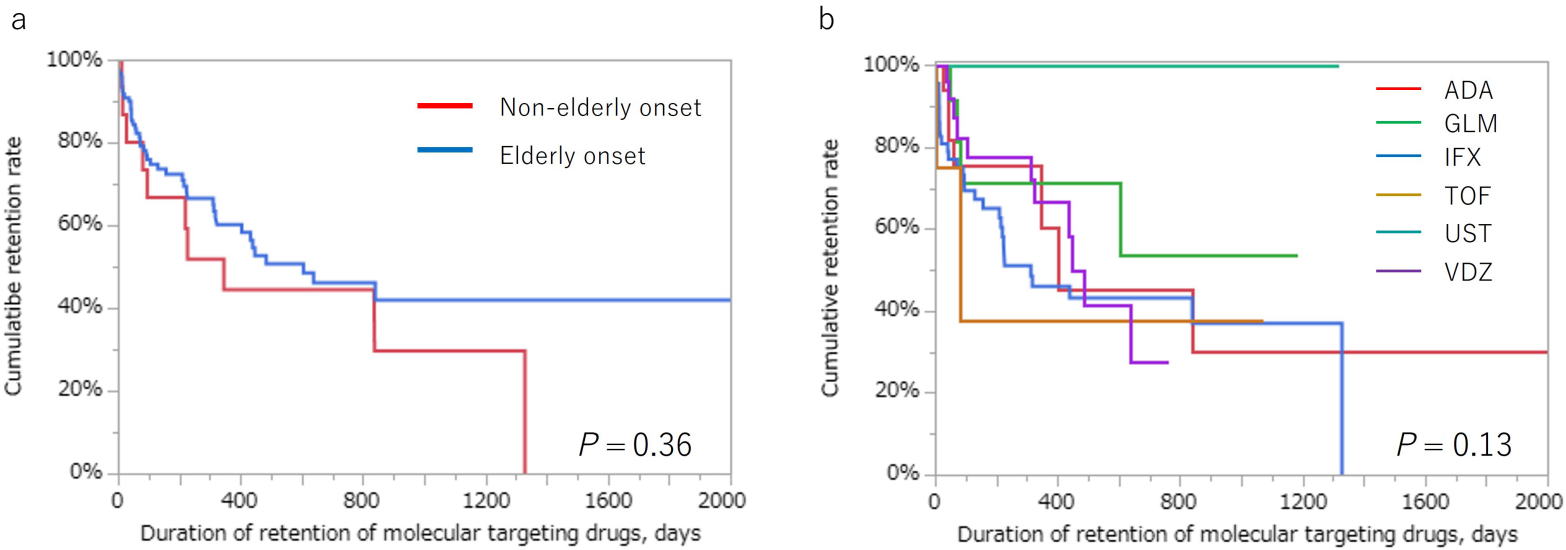
The cumulative treatment persistence rates of the first molecular targeting drug. (a) The cumulative treatment persistence rate of the first molecular targeting drug between elderly‐ and non‐elderly‐onset ulcerative colitis was not different (*P* = 0.36). (b) The cumulative treatment persistence rate of each drug in elderly patients with ulcerative colitis is not different (*P* = 0.13).

**Figure 4:**
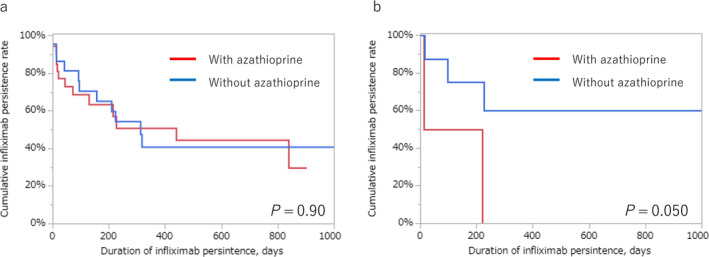
The cumulative infliximab persistence rates compared with and without azathioprine. (a). In the EO group, no significant difference was observed in the cumulative infliximab persistence rate between patients receiving infliximab with and without azathioprine (*P* = 0.90). (b) In the NEO group, no significant difference was observed in the cumulative infliximab persistence rate between patients receiving infliximab with and without azathioprine (*P* = 0.050).

In patients who received MTDs, no difference was observed in the cumulative treatment persistence rate of the first MTDs between the EO and NEO groups (Fig. 3a). The cumulative treatment persistence rates for each MTD are shown in Figure 3b. There was no difference in the treatment persistence days among each MTD (*P* = 0.13). Figure 4 illustrates the outcomes of comparing the combination therapy comprising infliximab and azathioprine with infliximab monotherapy. In the EO group, no significant difference was observed in the cumulative infliximab persistence rate between patients receiving infliximab with and without azathioprine (*P* = 0.90, Fig. 4a). Similarly, in the NEO group, a similar result was obtained (*P* = 0.050, Fig. 4b).

### Multivariate analysis for systemic steroid, MTDs, surgery, and death

EO was a clinical factor that increased the risk of systemic steroid administration (odds ratio [OR] = 1.38, 95% confidence interval [CI]: 1.10–1.73, *P* = 0.0048) and death (OR = 6.18, 95% CI = 1.52–25.15, *P* = 0.011), whereas EO was not a clinical factor that increased the risk for use of MTDs and surgery. Furthermore, systemic steroid administration associated with the risk of death (OR = 1.65, 95% CI = 1.13–2.41, *P* = 0.0091; Table 3).

**Table 3:** Multivariate analysis^†^ of the association among clinical factors and clinical events in patients with ulcerative colitis

Clinical factors	Number of patients *n* = 2946	Systemic steroid administration			Use of molecular targeting drug			Surgery		
		Hazard ratio	95% CI	*P*‐value	Hazard ratio	95% CI	*P*‐value	Hazard ratio	95% CI	*P*‐value
Sex	Male: 1664	Reference		0.94	Reference		0.43	Reference		0.15
	Female: 1282	1.00	0.89–1.14		0.89	0.67–1.19		0.61	0.31–1.20	
Age categories of UC onset	Elderly: 2669	**1.38**	**1.10–1.73**	**0.0048**	1.14	0.71–1.84	0.59	0.92	0.32–2.67	0.88
	Non‐elderly: 277	Reference			Reference			Reference		
Academic hospital	Yes: 16	**2.08**	**1.12–3.88**	**0.021**	2.56	0.95–6.90	0.063	**211.36**	**98.35–454.21**	**<0.0001**
	No: 2930	Reference			Reference			Reference		
Use of systemic steroid	Yes: 1008				**9.14**	**6.27–13.30**	**<0.0001**	0.89	0.42–1.87	0.75
	No: 1938				Reference			Reference		
Use of molecular targeting drug	Yes: 205							**2.30**	**1.03–5.14**	**0.043**
	No: 2946							Reference		

^†^ Cox proportional hazard model.

The bold value means statistical significance.

CI, confidence interval; UC, ulcerative colitis.

## Discussion

This study revealed that the use of systemic steroids, MTDs, and surgery rates did not differ between the EO and NEO groups; however, mortality rate in the EO group was higher than that in the NEO group. Although the cumulative systemic steroid‐free rate in the EO group was higher than that in the NEO group, the cumulative MTD‐free and surgery‐free rates between the EO and NEO groups did not differ. The cumulative treatment persistence rate of the first MTD rate also did not differ between the EO and NEO groups. Multivariate analysis demonstrated that EOUC was a clinical factor that increased the risk of systemic steroid administration and death.

Based on our findings, there may not be a significant variance in disease severity between EOUC and NEOU. Our investigation revealed that the cumulative systemic steroid‐free rate in the EO group was significantly lower than that in the NEO group, whereas the use of systemic steroids, MTDs, and surgery rates did not differ between the two groups. Moreover, prescription days of systemic steroid or the number of MTD use between the EO and NEO groups did not differ. Additionally, the cumulative infliximab persistence rate did not differ regardless of concurrent azathioprine in both EO and NEO groups. These outcomes collectively suggest a comparable level of disease severity between the two groups. Previous studies investigating the differences between EOUC and NEOUC have reported contradictory results. Several studies have demonstrated that NEOUC has more severe disease activity than EOUC,^23^, ^24^, ^25^ whereas one study showed that the rate of severe colitis during the observation period did not differ between EOUC and NEOUC.^8^ A topical review by the European Crohn's and Colitis Organization also stated that the severity of EOUC and NEOUC was almost similar.^26^ However, the definitions of elderly in these studies differed. Further prospective studies are warranted to determine whether disease activities of EOUC and NEOUC differ.

Our study also implies that patients with EOUC can avoid surgery using MTDs. Although multivariate analysis revealed that the use of MTDs was a clinical factor that associated with the risk of surgery, surgery and cumulative surgery‐free rates did not differ between the EO and NEO groups. A Japanese cohort study reported that patients with EOUC had a higher surgery rate than those with NEOUC.^27^ Our results may differ from those of this previous study because of the difference in NEOUC definition. Our study investigated the difference between EOUC patients and aged patients with NEOUC (patients who developed UC at age <65 years and became ≥65 years). In contrast, the previous study^27^ compared EO (patients who developed UC at age ≥65 years) and younger onset (all patients who developed UC at age <65 years) UCs. Therefore, the number of patients with NEOUC in our study was smaller compared with that of the previous study conducted in Japan.^27^ Although our study followed up one patient for approximately 4–5 years on average, the difference in surgery rates between EOUC and NEOUC might not have been recognized without a long observation period. A previous review concluded that the indications for surgery in older patients with UC were not different from that of non‐older patients with UC^26^. Therefore, further investigations on surgery rates differences between EOUC and NEOUC in longer observation periods are warranted.

We observed that the clinical use of MTDs did not differ between the EO and NEO groups, and no significant differences in the number of MTDs administered were observed. Although the breakdown of the first MTD did not differ between the EO and NEO groups, vedolizumab was more frequently selected in the EO group than in the NEO group, whereas infliximab was less frequently selected in the EO group than in the NEO group. Generally, older patients undergoing immunosuppressive therapies easily develop infections and complications due to frailty.^26^, ^28^, ^29^ One retrospective study reported that older patients with UC who were administered infliximab or adalimumab had a higher rate of severe infections and mortality compared with younger or older patients who were not treated with biologics.^30^ Another study revealed that oral corticosteroids increased the risk of serious infections.^31^ Contrastingly, a subanalysis of a phase III trial demonstrated that the safety of vedolizumab in older patients with UC was similar to that in other age groups.^32^ Therefore, physicians in Japan may select low immunosuppressive therapy for older patients compared with younger patients.

In this study, we observed that the EO group had a higher mortality rate than the NEO group. Our results showed that the average age at the end of observation period of EO groups was higher than that of NEO group (Table 2). Older age itself could contribute to high mortality rate because of various reasons including malignancy and cardiopulmonary diseases. However, the reasons of death in this study were unclear due to the nature of the DeSC database. Another study also reported a high mortality rate similar to our results.^33^ As described above, older patients are more likely to develop infections and complications, which contribute to a high mortality rate. Furthermore, a retrospective study from Japan showed high mortality rate (26.7%) after urgent surgery in older patients with UC.^34^ Thus, we suggest that the patient's condition and appropriate timing of surgery should be considered in older patients with UC. Elective surgery should be considered to the greatest extent possible, even if surgery is inevitable.

This study had some limitations. First, due to the nature of the DeSC dataset, it does not contain detailed clinical information of patients, including blood test data, endoscopic findings, disease extent, and computed tomography, which is necessary to determine the severity of UC. Therefore, we indirectly evaluated disease severity by investigating the rates of systemic steroids, MTDs, and surgery. For the same reason, we could not identify the reason of death. Second, although the DeSC dataset contains a large number of patients, its representativeness is not guaranteed because the rate of academic hospitals is remarkably low. Third, this was a retrospective study. A nationwide prospective study is required to comprehensively evaluate the differences between the EO and NEO groups. Despite these limitations, our analysis revealed differences between EOUC and NEOUC in a large dataset of patients with UC. Our findings are expected to enhance daily clinical practice for managing UC in the elderly and inform future investigations.

In conclusion, the severity and clinical course between the EOUC and NEOUC groups may not significantly differ in the long term after UC onset. The clinical practice for treating UC does not differ between the EOUC and NEOUC groups. However, we need to pay attention to the patient's condition and consider the appropriate timing of surgery when treating older patients with UC because of the high mortality rate in the EOUC groups. Further prospective investigations are warranted to confirm our findings.

## Data Availability

The corresponding author has opted to not share data because of a contract with DeSC Healthcare, Inc.
